# Positive Emotion and Honesty

**DOI:** 10.3389/fpsyg.2021.694841

**Published:** 2021-07-01

**Authors:** Evelyn Medai, Charles N. Noussair

**Affiliations:** ^1^School of Law, New York University, New York, NY, United States; ^2^Eller College of Management, University of Arizona, Tucson, AZ, United States

**Keywords:** honesty, happiness, virtual reality, emotion induction, experiment

## Abstract

We report an experiment that considers the impact of emotional state on honesty. Using the die-rolling task created by Fischbacher and Föllmi-Heusi to detect the level of dishonesty in a sample of individuals, we study the effects of induced happiness on the incidence of self-interested lying. The experiment uses 360-degree videos to induce emotional state. We find that people behave more honestly in a state of happiness than they do in a neutral state.

## Introduction

Individuals differ from each other in their propensity to behave dishonestly. Indeed, the same person may behave with more integrity on 1 day than another. What causes some people to behave more dishonestly than others? Factors such as background, personality, decision history, managerial philosophy, and reinforcement have all been shown to correlate with ethical behavior in business settings (Stead et al., 1990; see Ayal and Gino, 2011, for a survey). The payoffs at stake also exert an effect (Gneezy, 2005; Gneezy et al., 2020). But do *emotional states* also have an impact on honesty? If so, an organization might be able to use an insight in this regard to reduce unethical behavior by creating an environment conducive to particular emotional states. Since emotional states are malleable, interventions to do so may be feasible and cost-effective. The relationship between emotional state and honesty is the focus of the study reported here.

We report an experiment designed to explore the specific relationship between a positive emotional state and honesty. Our research question is whether individuals in a positive emotional state are more honest than those in a control treatment. To measure honesty, we utilize the die-rolling task created by Fischbacher and Föllmi-Heusi (2013). The task involves asking an individual to roll a die privately and then to report what was rolled. The individual receives a monetary payment based on the number that she reports. A player can typically earn more money if she makes a false report, and her actual roll can never be verified. Dishonest behavior can only be observed at the level of a sample, and not at the level of the individual^1^. In their original experiment, Fischbacher and Föllmi-Heusi (2013) found that individuals lie on average, but not to the maximum extent possible. Typically, a sample of individuals exploits on average ¼ of the potential monetary gains from lying (Abeler et al., 2019).

The die-rolling task has become standard in experimental economics to measure honesty, and follow up studies confirm the existence of substantial, though less than ubiquitous, dishonesty. Abeler et al. (2019) have reviewed 90 studies using the Fischbacher and Föllmi-Heusi paradigm to identify the correlates of truth-telling. Among the patterns that they report, they find that women behave more honestly than men on average, and that among student participants, field of study has no effect on honesty. Rosenbaum et al. (2014), in an earlier survey of 63 experiments on ethical behavior, similarly report that there is evidence that women behave more ethically than men on average, and that the results on whether economics and business students differ from others are mixed. These findings are relevant to our work in that we test for and find no difference between the genders or between business/economics majors and those enrolled in other programs of study. None of the studies that Rosenbaum et al. (2014) or Abeler et al. (2019) reviewed studied the causal relationship between honesty and emotional state.

There has been some previous work on the connection between other emotions and unethical behavior. Motro et al. (2018) find that anger increases, while guilt reduces, deceptive behavior. Klygte et al. (2013) also report that anger leads to less ethical, while fear induces more ethical, decisions. Lim et al. (2015) find that subliminal priming with disgusted faces makes individuals slightly more honest in a mind-game die-rolling task (Jiang, 2013),^2^ which is closely related to the task we employ. Kugler et al. (2021) find no relationship between disgust and honesty in three different tasks. Brain imaging studies have revealed a network of brain regions that exhibit greater activation when individuals are being deceptive, suggesting that lying is more demanding of the brain than honesty (see e.g., Greene and Paxton, 2009). We are unaware of any research studying the causal impact of happiness on ethical behavior.

The experiment reported in this paper has two treatments: one in which a positive emotional state, which we will refer to as *Happiness*,^3^ is induced, and one control treatment, which we call *Neutral*. As a means of emotion induction, we employ a novel method. We conduct the experiment using Oculus Rift virtual reality headsets, which participants use to view a 360-degree video that induces happiness or one that does not have an effect on emotional state.^4^ After subjects watch the video, they are sent into another room where they are read a set of instructions that describe how they are to roll the die, and are informed that the die roll would be completely private. They are then sent out of the room one at a time to roll the die privately out of the view of any other person, and to report their roll to an experimenter in another room.

We find that the Happiness treatment results in lower levels of dishonesty than the Neutral treatment. The effect is significant at conventional or borderline levels, depending on the statistical analysis that is employed. We observe no significant difference in lying between women and men. Section Experimental Design describes the experiment and section Results reports the results. We offer some concluding remarks in section Conclusion.

## Experimental Design

### Procedures Common to All Treatments

This experiment is an individual decision-making task, with subjects acting completely independently of each other. The study was conducted with 106 University of Arizona students between November 2017 and May 2018, with 53 participants assigned to each of two treatments. Between three and six subjects participated in each session. All sessions were conducted at the Economic Science Laboratory at the University of Arizona, located in Tucson, Arizona, USA. There were no other tasks conducted in the session, either before or after those described here. The subjects were recruited from the laboratory's subject pool and were all undergraduates from a variety of programs at the university. Of the 106 total subjects, 48 were male and 58 were female. Sixty-five were studying economics or business and the remaining 41 were pursuing other studies.

At the beginning of a session, subjects reported to Room A, one of the rooms in the Economic Science Laboratory facility. The experiment began with individuals watching a video using an Oculus Rift virtual reality headset in Room A for ~5–6 min. These videos were played using a program called Virtual Desktop and induced either a state of Happiness or one of Neutrality. The videos are filmed from a perspective of someone inside the video and displayed in 360 degrees. This means that the subject sees the video no matter in which direction she is looking and feels like an active participant in the video. The experience is highly immersive.

After the video, subjects were led to Room B, another room in the laboratory facility adjacent to room A, where an experimenter read the instructions for the die-rolling task. The subjects also had a written copy of the instructions they could use to follow along [a copy of the instructions can be found in the Appendix (Supplementary Material)]. These instructions were very short so that the effect of the induced emotion did not have time to dissipate. They explained to participants how they were to roll a six-sided die and report their roll to the experimenter. The instructions also explained that the subject would be the only one to observe the die roll. They also indicated how subjects would be paid. In addition to a $2 fee paid to all participants for viewing a video, a subject was given $2 times the number of the die roll that she reported. Therefore, in addition to the $2 payment for watching the video, subjects received $2 if they claimed that they rolled a 1, $4 if they reported a 2, $6 if they indicated a 3, and so on. The higher the reported roll, the higher the payoff that the subject received. Since the roll was entirely private, with no other participant or experimenter ever knowing the true result of an individual roll, subjects had a material incentive to lie.

While it was impossible to know which individual subjects were lying, collecting many observations of data reveals the average level of dishonesty in a group. In any group, if all subjects are honest, the result would be an approximately uniform distribution of the frequency of reports of each number on the die. With a six-sided die, each number should make up ~16.667% of the total number of observations. Lying causes the distribution of frequencies to shift, and if the lying is self-interested, it would shift toward higher numbers. The average report, and the percentage of individuals submitting the highest-paying report, can be interpreted as measures of the extent of self-interested lying among participants in a given treatment.

Once the instructions were read, subjects were sent out of room B, one-by-one, to roll their die. The die roll was completely private, with no experimenters or other participants witnessing the roll. The subjects were instructed that they could go anywhere in the building to perform the task, and that they should make sure to roll the die privately. They then returned to either Room A or another available, empty room (depending on the session) where an experimenter was present. The subject reported the roll to the experimenter with the room door closed and was paid accordingly. They were then asked to immediately leave the building. Each subject was sent out of room B to roll the die only after the previous participant had completed reporting her roll in the other room and had left the area. These procedures ensured that no other participants were within view or earshot when a report was made, and that subjects could not discuss their reports with each other before submitting them. In addition to the number rolled on the die, subjects were asked about their major (program of study) after they reported their roll. The experimenter also recorded their gender.

### Treatments

Subjects were shown one of two 360-degree videos in virtual reality, depending on the treatment. The experimental design had a between-subject structure, in that each subject was only shown one video and performed the die-rolling task only once. No subject participated in the experiment more than once. Of the male participants, 25 were in the Neutral treatment and 23 were in the Happiness condition. Twenty-eight females were in the Neutral, and 30 took part in the Happiness, treatment respectively.

#### Neutral

the video for the control treatment was a simple video of a tulip field on a sunny day. The video is taken from the perspective of an individual sitting in the field. There was no music, but there were soft noises, such as birds chirping and distant chatter. The video lasted for ~5 min and subjects were shown the video once before proceeding with the rest of the experiment. The video can be found at https://www.youtube.com/watch?v=SmhuzTzUKQY.

#### Happiness

The video inducing a state of happiness was a video shown from the point of view of surfers in a tropical beach setting. Viewers would get a first-person viewpoint of surfing on waves, paddling out to sea, and swimming in the ocean. Accompanying the visual component of the video, there was upbeat, positive music playing, further promoting a pleasant experience. The video was approximately two and a half minutes long and subjects were shown the video twice; the video was immediately played again once it finished playing for the first time. This video was played twice to maintain consistency among video lengths across treatments. This video can be viewed at https://www.youtube.com/watch?v=MKWWhf8RAV8^5^.

We conducted a manipulation check during several earlier sessions, with different individuals than those who participated in the experiment, to verify that the videos increased the level of the targeted emotion while not increasing any others. In the manipulation check, we asked individuals to report the levels of five emotions: Happiness, Fear, Sadness, Anger, and Disgust, that they were currently experiencing. We asked these participants to indicate, on a scale of 1–7, the strength with which they felt each emotion. They did so both before and after viewing one the videos. The data are given in the Table 1.

**Table 1:** Manipulation check: average self-reported emotional states on a scale of 1–7, before and after viewing the videos.

**Average self-reported emotion**					
**Emotion condition**	**Disgust**	**Sadness**	**Happiness**	**Fear**	**Anger**
Before video (*n* = 47)	1.33	2.14	4.21	2.32	2.01
After Neutral video (*n* = 22)	1.15	1.33	4.55	1.4	1.14
After Happiness video (*n* = 25)	1.21	1.14	5.36	1.46	1.55

The table shows that the Neutral video did not increase the strength of any of the emotions (other than an insignificant increase in happiness). The Happiness video raised average reported happiness while not increasing any of the other emotions.^6^ The average level of self-reported happiness, 5.36, was significantly greater after viewing the Happiness video than before viewing a video (4.21). A pooled variance *t*-test rejects the hypothesis that the two means are equal (*t* = 1.89, *p* < 0.05). The Neutral video did not yield a level of self-reported happiness significantly different from that recorded prior to the viewing of a video (*t* = 0.515, *p* > 0.25). Those who viewed the Happiness video reported a greater degree of happiness afterward than those who had viewed the Neutral video (*t* = 1.31, *p* < 0.1), though the effect is only borderline significant. The average level of each of the other four emotions after viewing a video is not different between the two treatments.

### Hypothesis

Before conducting the experiment, we formulated the following hypothesis regarding our treatment differences. Since there are no prior results, to our knowledge, to guide our a priori beliefs, we have no basis to hypothesize a sign for a treatment effect. Thus, our hypothesis is a two-sided claim that there would be no treatment effect.

*Hypothesis: People behave equally honestly in the Happiness and Neutral treatments. The average reports, as well as the percentage of individuals reporting a roll of 6, are not different between the two treatments*.

For a two-sided *t*-test of the hypothesis that there is no difference in average report between treatments, our sample size yields a power of 73% to detect a medium sized treatment effect of 0.5 standard deviations at a significance level of 0.05. In terms of the proportion of individuals claiming a roll of 6, our sample size yields a power of 60% of detecting a difference between treatments at a significance level of 0.05, if the true means are 0.17 and 0.35 in the two treatments. Although the experiment was not designed specifically to do so, in our analysis of the data, reported in section Results, we also consider whether there are differences in the level of honesty between women and men, and between economics/business majors and those pursuing other programs of study.

## Results

### Summary of Data

**Figure 1:**
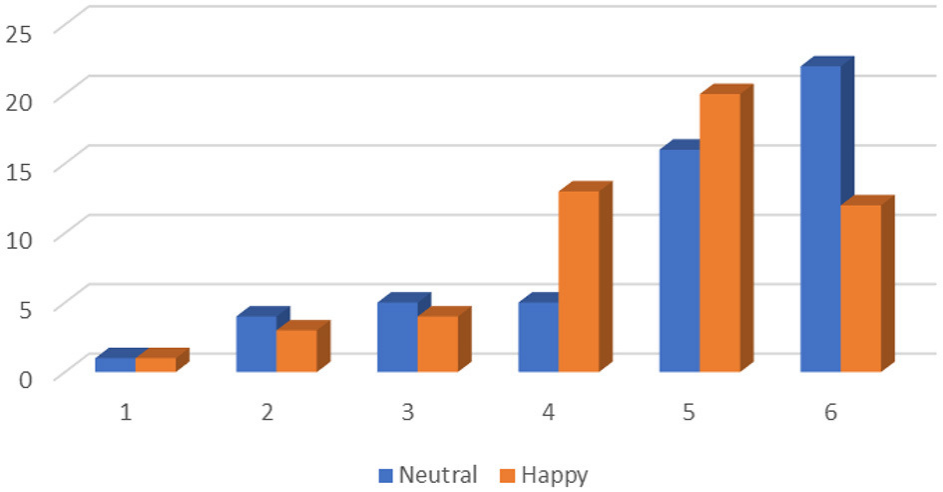
Reported rolls, both treatments.

The distribution of reported dice rolls in each treatment can be seen in Figure 1. In the figure, the vertical axis represents the number of individuals who reported a particular roll, while the horizontal axis indicates the roll reported.

In the Neutral treatment, the average report was 4.83 (std. dev = 1.369), significantly greater than the average under honest reporting of 3.5 (*t* = 7.07, *p* < 0.001). There was also a greater than random incidence of the reporting of 5 or 6. Thirty-eight of the 53 subjects reported rolling either a 5 or a 6. If people had been honest, we would expect 17.67 out of 53, one-third of, subjects to report either a 5 or a 6. We reject the hypothesis that the proportion reporting 5 or 6 is equal to 1/3, using a binomial test (*z* = 5.92, *p* < 0.001). On the other hand, if all participants were selfish and willing to be as dishonest as needed to maximize their monetary payment, all players would report a six. This is also clearly not observed, with only a minority of participants reporting a 6. Gender differences are small and insignificant, with 40% of men and 42.8% of women reporting a 6, and the average reports being 4.89 and 4.76 for women and men, respectively.

In the Happiness treatment, the average reported roll was 4.58 (std. dev = 1.20), 0.25 lower than in the Neutral treatment. This is also significantly different from the average under honesty of 3.5 (*t* = 6.55, *p* < 0.01). Figure 1 shows that 32 of 53 subjects reported a 5 or a 6, significantly greater than under honest reporting (*z* = 4.17, *p* < 0.01). However, only 12 subjects in the Happiness treatment reported rolling a 6 (22.6%). In the Happiness treatment, 20% of women and 26.6% of men claimed a six, and the average report was 4.5 and 4.69 for women and men, respectively. The difference in the average report between the two treatments is therefore 0.39 for women and 0.06 for men. There is a 18.9% point difference between treatments in the incidence of claiming a roll of 6, with almost twice as many claims of 6 in the Neutral treatment. Because women make slightly lower average reports than men under the Happiness treatment, while making slightly higher reports than men under Neutral, there is no overall gender effect.

### Formal Comparison Between Treatments

We conducted a number of formal statistical tests to compare the average report, the incidence of extreme lying, and the distribution of reports, between the two treatments. A *t*-test comparing the difference in means between the Neutral and Happiness treatments results in a *t*-statistic of 2.16, significant at *p* < 0.05 (two-tailed test). The Neutral treatment generates a higher average report than the Happiness condition.

To compare the amount of extreme lying between treatments, we conduct a binomial test of the hypothesis that the proportion of 6s is equal in the two treatments. The test yields a *p*-value of 0.038. The probability of claiming 6 is significantly lower in the Happiness than in the Neutral treatment.

Finally, we conducted a chi squared test to determine whether there were significant differences in the distribution of reported rolls between treatments. This test results in a statistic of 18.795. At five degrees of freedom, this is significant at 1%. Thus, the distribution of reports differs between the two conditions.

### Regression Analysis

To evaluate the hypothesis, while controlling for influences that might affect the comparison between treatments, we conducted regressions with two different dependent variables. The first is a dummy variable, which takes on a value of 1 if the participant rolls a 6 and 0 otherwise. These regressions consider the determinants of extreme lying. The second is the actual reported roll, a measure of the general tendency to lie. The dummy variable *Happiness* was coded as a 1 for the Happiness treatment and 0 otherwise. To create the variable “*Major*,” business and economics majors were coded as a 1 and all other majors were coded as a 0. For the “Gender” variable, all males were coded as a 1 while all females were coded as a 0.

In Table 2, the estimates for the variable Happiness reveal an effect of treatment that is significant at *p* < 0.1, and that is robust to the specification. It confirms, albeit at a marginal significance level, that controlling for gender and major, there is more honest behavior in the Happiness than in the Neutral treatment. Splitting the sample between women and men, however, reveals that the treatment effect is specific to women. The variable Happiness is significant in equation (4) though not in (5). In specifications (1)–(3) in which both women and men are included, the coefficient for Happiness is marginally significant, indicating that there is a treatment effect for women (the base category for gender). However, the sum of the coefficients for Happiness and Gender^*^Happiness is not significant, indicating that there is no treatment effect for men.

**Table 2:** Determinants of claiming a six and of overall roll claimed.

	**Prob. claim 6 (Probit) (1)**	**Prob. claim 6 (Logit) (2)**	**Claim (OLS) (3)**	**Claim (OLS) women only (4)**	**Claim (OLS) men only (5)**
Constant	−0.354 (0.349)	−0.513 (0.577)	5.144^***^ (0.346)	5.352^***^ (0.401)	4.741^***^ (0.416)
Happiness	−0.549^*^ (0.360)	−0.916^*^ (0.607)	−0.458^*^ (0.346)	−0.480^*^ (0.336)	−0.015 (0.403)
Gender	0.032 (0.351)	0.005 (0.567)	−0.173 (0.358)		
Major	0.096 (0.297)	0.161 (0.497)	−0.293 (0.285)	−0.587^*^ (0.381)	−0.047 (0.432)
Gender × Happiness	0.168 (0.523)	0.295 (0.869)	0.374 (0.508)		
*n*	106	106	106	58	48

*The dependent variable in columns (1) and (2) is a dummy variable that equals 1 if an individual reports a roll of 6, and 0 otherwise. The dependent variable in columns (3)–(5) is the die roll an individual reports. Column (1) is a Probit specification, (2) is a Logit, and (3)–(5) are OLS specifications. Each individual is an observation. n = 106*.

* *Means p < 0.1,*

*** *Refers to p < 0.01, standard errors in parentheses*.

The regressions also show that there is no overall effect of gender on honesty. There is also no effect of program of study for the sample as a whole. However, there is an effect of major if only women are considered. Women who are studying business or economics submit lower reported rolls than those pursuing other majors.

## Conclusion

We observe some evidence that people are more honest in a state of happiness than in a state of neutrality. In the laboratory, emotions can have an effect on the extent of ethical behavior. We do not know, for now, how general this relationship is. However, if the effect transfers to a workplace environment, it would indicate that a creating a more positive workplace environment would lead to more honest behavior on the part of employees. Research on how to create a positive workplace culture is well-developed. Some of these strategies include caring for colleagues on a personal level, providing support and compassion when others are struggling, avoiding blame, forgiving mistakes, and emphasizing the meaning of the work being done (Seppala and Cameron, 2015). Using such techniques to create a positive work environment may lead to a decrease in dishonesty in the workplace. Similarly, if schools and universities are able to improve overall levels of positive emotion in students, particularly when they are in the classroom, it could lead to a reduction in academic dishonesty.^7^

We observed no significant difference in honesty by gender. The conclusion that there was not a significant effect of gender on honesty provides yet another rationale for the equal treatment of the genders in the workplace. There is no reason to believe, based on what we have observed in this study, that an employee or a student of one gender would be more or less ethical than an individual of another gender.

## Data Availability Statement

The raw data supporting the conclusions of this article will be made available by the authors, without undue reservation.

## Ethics Statement

The studies involving human participants were reviewed and approved by Institutional Review Board of the University of Arizona. The patients/participants provided their written informed consent to participate in this study.

## Author Contributions

Both authors were involved in designing and conducting the experiment, analyzing the data, and writing the manuscript.

## Conflict of Interest

The authors declare that the research was conducted in the absence of any commercial or financial relationships that could be construed as a potential conflict of interest.

## Footnotes

^1^As an alternative task, one could also employ the version of the dice-rolling task used by Pascual-Ezama et al. (2020). In their version, participants roll a die electronically on a website provided by the experimenter and are asked to enter the result on their computer. The result of the roll is recorded so that the experimenter can know if a roll actually took place, and can compare the result to the participant's report. This would allow the experimenter to identify different types of dishonest behavior: misreporting the roll, not rolling the die at all, and rolling multiple times until a favorable result is achieved.

^2^In the mind game task studied by Jiang (2013), individuals are told to role a die “in their minds,” that is, to imagine a die roll that does not actually physically take place, and then to report the outcome of the roll.

^3^We recognize that “Happiness” is a broad term, with a variety of uses and meanings in the scientific literature. We use the term here to describe our treatment condition as a concise term to describe the positive emotional state that is induced by our video. We recognize that positive emotional state is only one component of subjective well-being. For example, Seligman (2011, 2018) considers positive emotional state as one of the five dimensions in his well-known PERMA model of well-being. Our pretest results show that individuals do describe themselves as “happier” after viewing the video than they were before viewing it.

^4^There is a long tradition of using videos to induce emotional state. Using 360 videos shown in virtual reality, in our view, constitutes a more intensive implementation of this established method. See Kugler et al. (2020) for an example of the use of virtual reality to induce emotional state for participants in a trust game.

^5^There are obviously many types of positive emotions, and among these are a number of states that are referred to with the term “Happiness.” Sports tend to create positive emotional states with high arousal. See Hills and Argyle (1998) for a discussion of this point. It is quite possible that different types of positive emotional state may exert different effects on honesty, and this is an agenda of questions that can be addressed in follow-up research. Hills and Argyle show that participating in sports increases positive feelings toward others and toward life, improves body image and self-esteem, and creates feelings of achievement and excitement. Any of these could serve as channels whereby the surfing video, which simulates a sporting activity in virtual reality, might increase or decrease honest behavior. Nevertheless, Hills and Argyle found no correlation between participation in sport and scores on a social conformity index that they interpret as a “lie scale”.

^6^The Happiness video lowered the average level of sadness (*t* = 2.46, *p* < 0.05) and fear (*t* = 2.77, *p* < 0.01) significantly, but did not significantly affect the average level of disgust or anger. The Neutral video reduced the levels of sadness (*t* = 3.19, *p* < 0.01), fear (*t* = 2.47, *p* < 0.05), and anger (*t* = 3.23, *p* < 0.01) significantly.

^7^It may be that such relatively early inducement of changes in habits might have long-term effects; studies have found that college students who engage in academic dishonesty are more likely to act dishonestly in the workplace (Nonis and Swift, 2001).
