# Supplementary material for: Positive Emotion and Honesty
Source: Front Psychol. 2021 Jul 1;12:694841. doi: 10.3389/fpsyg.2021.694841 (PMC8281290; doi:10.3389/fpsyg.2021.694841)
Supplement: Supplementary file 1 [file Data_Sheet_1.docx]

**Supplemental material for “POSITIVE EMOTION AND HONESTY”**

**Appendix A: Experiment Instructions**

Instructions for Experiment

In this experiment, you determine your payoff by rolling a die. Your die roll decides how much money you receive for this experiment. You receive $2 times the number you roll on the die. Therefore, you receive $2 if you roll a 1, $4 if you roll a 2, $6 if you roll a 3, $8 if you roll a 4, $10 if you roll a 5, and $12 if you roll a 6. In addition to what you get from your die roll, you receive a $2 show-up fee for participating in the experiment.

You will roll the die privately. We will call you up one at a time, and give you the die. Please then leave the room and go somewhere of your choosing in this building to roll the die. Please then come to room 112, report what you rolled, and return the die. You must come to room 112 within two minutes. We will then pay you your earnings. When you come to room 112, only you and an experimenter will be present. No other participant will ever know what your die roll was.

**Appendix B. Data**

| Neutral Treatment | | | |
| --- | --- | --- | --- |
| Participant number | Gender | Major | Reported Roll |
| 1 | Female | Business Economics | 6 |
| 2 | Male | Business Management | 6 |
| 3 | Male | PPEL | 1 |
| 4 | Male | Chemical Engineering | 5 |
| 5 | Male | Economics | 6 |
| 6 | Male | Urban & Regional | 6 |
| 7 | Female | MIS | 4 |
| 8 | Male | Economics | 4 |
| 9 | Female | Accounting | 6 |
| 10 | Female | Finance | 5 |
| 11 | Female | Pre-Business | 4 |
| 12 | Female | Pre-Business | 5 |
| 13 | Female | Business | 2 |
| 14 | Male | Anthropology | 5 |
| 15 | Male | Accounting | 5 |
| 16 | Female | Economics | 6 |
| 17 | Female | Economics | 6 |
| 18 | Male | MIS | 2 |
| 19 | Male | Accounting | 5 |
| 20 | Female | Economics | 5 |
| 21 | Female | Pre-Business | 6 |
| 22 | Female | Marketing | 5 |
| 23 | Female | Marketing | 3 |
| 24 | Male | Biology | 6 |
| 25 | Male | Pre-Business | 4 |
| 26 | Female | Marketing | 6 |
| 27 | Female | Retail and Consumer Sciences | 3 |
| 28 | Female | Political Science | 5 |
| 29 | Female | Business | 3 |
| 30 | Male | MIS | 6 |
| 31 | Female | Systems Engineering | 3 |
| 32 | Male | Business | 5 |
| 33 | Female | Management | 3 |
| 34 | Female | Business | 5 |
| 35 | Female | Retail and Consumer Sciences | 5 |
| 36 | Male | Marketing | 4 |
| 37 | Male | Business Management | 6 |
| 38 | Male | Business | 6 |
| 39 | Female | Pre-Business | 5 |
| 40 | Male | Business Economics | 2 |
| 41 | Female | Management and Entrepreneurship | 6 |
| 42 | Male | Pre-Business | 2 |
| 43 | Female |  | 6 |
| 44 | Female | Accounting | 6 |
| 45 | Male | Pre-Business | 5 |
| 46 | Male | Architecture | 6 |
| 47 | Male | Economics | 6 |
| 48 | Female | Environmental Studies | 6 |
| 49 | Male | Marketing | 6 |
| 50 | Male | Electrical Engineering | 5 |
| 51 | Female | Finance | 6 |
| 52 | Male | Engineering | 5 |
| 53 | Female | Pre-Business | 6 |
| Happiness Treatment | | | |
| Participant number | Gender | Major | Reported Roll |
| 54 | Female | Agriculture | 5 |
| 55 | Female | Business Management | 5 |
| 56 | Male | Regional Development | 3 |
| 57 | Female | Pre-Business | 5 |
| 58 | Female | Marketing | 5 |
| 59 | Female | Political Science | 4 |
| 60 | Female | Business Economics | 3 |
| 61 | Female | Family Studies & Human Development | 4 |
| 62 | Male | Finance | 5 |
| 63 | Male | Japenese | 2 |
| 64 | Male | Economics | 6 |
| 65 | Male | Economics | 5 |
| 66 | Female | Accounting | 4 |
| 67 | Female | Pre-Business | 4 |
| 68 | Female | Marketing | 4 |
| 69 | Male | Chemical Engineering | 5 |
| 70 | Male | Economics | 5 |
| 71 | Female | Communications | 4 |
| 72 | Female | MIS | 4 |
| 73 | Male | Political Science | 4 |
| 74 | Female | Pre-Business | 5 |
| 75 | Female | Pre-Business | 6 |
| 76 | Female | Mechanical Engineering | 5 |
| 77 | Male | Mathematics | 5 |
| 78 | Male | Nutrition | 5 |
| 79 | Male | Pre-Business | 5 |
| 80 | Male | Business Economics | 6 |
| 81 | Male | Physiology | 5 |
| 82 | Female | Marketing and MIS | 1 |
| 83 | Male | Entrepreneurship | 5 |
| 84 | Female | Biology | 5 |
| 85 | Male | History | 4 |
| 86 | Male | Business Management | 2 |
| 87 | Male | Finance | 6 |
| 88 | Male | Pre-Business | 4 |
| 89 | Female | Accounting | 4 |
| 90 | Male | PPEL | 6 |
| 91 | Female | MIS | 6 |
| 92 | Male | Economics | 4 |
| 93 | Male | Finance | 6 |
| 94 | Female | Retail and Consumer Sciences | 3 |
| 95 | Female | Pre-Business | 5 |
| 96 | Female | Geography | 6 |
| 97 | Female | Accounting | 5 |
| 98 | Female | Economics | 6 |
| 99 | Female | Marketing | 2 |
| 100 | Female | Elementary Education | 5 |
| 101 | Female | Elementary Education | 5 |
| 102 | Male | Accounting | 4 |
| 103 | Female | Psychology | 6 |
| 104 | Female | MIS | 3 |
| 105 | Male | Marketing | 6 |
| 106 | Female | Psychology | 6 |
